# The impact of the government response on pandemic control in the long run—A dynamic empirical analysis based on COVID-19

**DOI:** 10.1371/journal.pone.0267232

**Published:** 2022-05-04

**Authors:** Yuxun Zhou, Mohammad Mafizur Rahman, Rasheda Khanam

**Affiliations:** School of Business, University of Southern Queensland, Toowoomba, Queensland, Australia; Nanyang Technological University, SINGAPORE

## Abstract

**Purpose:**

Although the outbreak of the Corona Virus Disease 2019 (COVID-19) occurred on a global scale, governments from different countries adopted different policies and achieved different anti-epidemic effects. The purpose of this study is to investigate whether and how the government response affected the transmission scale of COVID-19 on the dynamic perspective.

**Methodology:**

This paper uses a dynamic generalized moment method to research the relationship between the government response and COVID-19 case fatality rate by using panel data from eight countries: China, United States, Canada, Australia, Italy, France, Japan, and South Korea.

**Findings:**

We have the following findings: 1. Government responses have a significant impact on the scale of COVID-19 transmission. 2. The rate of increase of government responses on the growth rate of COVID-19 case fatality rate has the characteristics of cyclicity and repeatability, that is, with the increase in the growth rate of government responses, the COVID-19 case fatality rate shows the following cyclical motion law: increasing first, reaching the maximum point, and then declining, and finally reaching the minimum point and then rising; ultimately, its convergence becomes 0. The cyclical fluctuations of COVID-19 in the long term may be caused by the decline in the level of government response, the mutation of the virus, and the violation of restrictive policies by some citizens. 3. The government response has a lag in controlling the spread of COVID-19.

**Originality/Value:**

Since there is a lack of literature on the impact of government responses on the development of COVID-19 from a long-term and dynamic perspective. This paper fills this gap in empirical research. We provide and expand new empirical evidence based on the current literature. This paper provides the basis for government decision-making and will help to formulate the response to other major public health events that may occur in the future.

## 1. Introduction

In early 2020, COVID-19, which originated in China, gradually but inexorably spread around the world. What had started as an epidemic evolved into a pandemic. COVID-19 is very easily transmitted, mainly through close contact with others, and particularly through airborne transmission [1], with a relatively high infection and mortality rate [2]. Governments around the world have adopted different degrees and types of response policies to control the spread of COVID-19. Different response policies in different countries correspond to different control effects. Countries such as Japan [3], South Korea [4], China [5], and Australia [6] have achieved the desired results through strict control policies. European countries (https://www.ecdc.europa.eu/en), some other Asian countries and South American countries originally achieved some level of control through stringent policies, but once they were relaxed, new outbreaks occurred.

Government response is the most important component of disaster management. [7] summarized disaster management in four parts: mitigation, preparedness, response, and recovery. "*The response phase of disaster relief happens right after a disaster occurs and refers to any activities that work to take care of the damage caused by the disaster*" [7]. Research on government response and disaster management involves seven aspects: 1. How to achieve the optimal allocation of resources [8]; 2. Project schedule [9, 10]; 3. The interaction between the government and the company [11]; 4. The evacuation process [12]; 5. The decision-making system [13]; 6. Network analysis [14–16]; 7. Supply chain optimization [17]. Therefore, the impact of response on disaster management is multidimensional. COVID-19 has also been a disaster for humans. Therefore, it is important to study the impact of the government response on the scale of the pandemic to reduce the losses caused by COVID-19. Unlike some public health emergencies in the past, COVID-19 is characterized by long duration and rapid spread. COVID-19 continues to appear around the world today. In contrast to the SARS virus, COVID-19 has not disappeared because of rising temperatures. Therefore, it has been postulated that humans will continue to struggle with COVID-19 for a long time. Consequently, based on the above background statement, it is important to investigate the relationship between government response and COVID-19 control over the long term. In the case of COVID, which continues to mutate, this is a dynamic relationship and is therefore more difficult to control.

There is a lack of literature on the impact of government response on the development of COVID-19 in the long-term from a dynamic perspective. To the best of our knowledge, no such studies are found in the existing literature. This is the main motivation for pursuing this research.

Against this backdrop, we propose the core research topic in this paper: the impact of the government response on pandemic control in the long run. We put forward the following two research questions:

*Q1*: Can the government response affect the spread of COVID-19 in the long run?
*Q2*: How has the government response affected the spread of COVID-19 in the long run?

The main contributions of this research are follows: First, we fill in the empirical evidence for dynamic pandemic research. We examine the dynamic process of government responses to the development of COVID-19. Based on the dynamic trajectory of COVID-19, we provide relevant policy recommendations. 2. Our findings will provide empirical evidence for management of the COVID-19 outbreak and help governments make appropriate decisions.

In the second section, we will briefly review the literature on COVID-19 and government response research. In the third section, we introduce an economic model and econometric methods. In the fourth section, we make an empirical analysis. In the final section, we conclude the paper with policy implications.

## 2. Literature review

There is a lack of relevant economic literature on COVID-19, as it is a new theme for research. We have searched extensively for literature on the issue of COVID-19 and government responses but failed to locate any research outputs on this topic. In this paper, we intend to fill this gap.

Existing literature on COVID-19 and government responses are all based on the Stringency Index of the University of Oxford. [18] used the index to study the driving factors of citizens’ satisfaction with government responses. They found that citizens are more concerned about the results of COVID-19 control than the policies the government had adopted. They also found that citizens’ satisfaction with the government’s response varies greatly from country to country. In conclusion, they analyzed a possible controversy: governments, in implementing possible exit strategies to escape the current lockdown, generally try to strike the right balance between the impact on public health (saving lives) and the impact on the economy (saving jobs). Some governments would prefer to reopen their markets to minimize the impact of the outbreak on their economies, while others are reluctant to take this course of action because it could lead to a rapid increase in infections. This is also one of the problems addressed by this paper. We will provide empirical evidence for this argument.

Delays in policy can lead to serious consequences. [19] studied the impact of policy decisions on mortality. They found that policy delays increased mortality (travel restriction policies, public advocacy policies, etc.). They believed that testing policies may also have an impact on mortality rates. An increase in the number of tests might reduce the death rate.

[20] studied the impact of restriction policies on COVID-19 in East Asia. They found that the incidence of COVID-19 decreased after the implementation of a non-pharmacological intervention. They judged that citizens’ responsibility, collectivism, and vigilance were the three main reasons for the success of COVID-19 in East Asia. They undertook a short-term analysis of the impact of the government response on COVID-19. However, the battle against COVID-19 is long- term. In the long run, whether the government response (restriction policy) is effective for epidemic control remains a gap in the literature. This is also one of the main problems analyzed in this paper.

Other studies have explained the informational impact of the government response on epidemic control. Before COVID 19 appeared, [21] had studied how a government should respond to a pandemic. They considered that the best response would be transparency and accountability. [22] researched the Philippine government’s response to the COVID-19 outbreak in the Philippines, demonstrating that transparency and good information delivery mechanisms are key to dealing with public events.

Combining the above research literatures on COVID-19 with the government response, we find that there is a lack of dynamic research on the long-term effects between the government response and COVID-19. Whether and how the government response will affect the development of the pandemic in the long term is a research gap in the existing literature.

## 3. Methodology

### 3.1 Theoretical model

We firstly made basic assumptions about the model. According to the research object of this paper: government response action and epidemic control, we first determined the independent variable (government response) and the dependent variable (epidemic control). We made assumptions about government and citizens, respectively. The government budget balance required the following equation:

$$G_{t}^{h}\cdot \psi_{t}=T_{t}-G_{t}^{f}+\theta_{t}$$ (3.1.1)

Where $G_{t}^{h}$ denotes government spending to contain the epidemic (the cost of restrictions such as travel bans, outdoor recreation bans etc.); ***ψ*** = (0,1) and if ***ψ*** = 0 represents no government restriction policies, ***ψ*** = 1 otherwise. *T*_*t*_ represents tax. $G_{t}^{f}$ denotes spending in addition to spending in relation to restriction policy. *θ*_*t*_ is random error term.

The representative household maximizes the expected value of the following utility function:

$$U({\left(J^{\psi }\right)}_{t}C_{t}^{O},S_{t}|G_{t}^{h}\psi )=C_{t}^{I}+H(E_{t},M_{t},F_{t},G_{t}^{h}\psi_{t};\vartheta )+{\left(J^{\psi_{t}}\right)}_{t}C_{t}^{O}+\epsilon_{t}^{J},\;\psi_{t}=0,1;i=0,1$$ (3.1.2)

Where instantaneous utility function *U*(⋅) has three independent variables: indoor consumption, $C_{t}^{I}$, outdoor consumption ${\left(J^{\psi_{t}}\right)}_{t}C_{t}^{O}$ and health level *H*, which is a latent variable. We assume that Covid-19 is intertemporal. *E*_*t*_, *M*_*t*_, *F*_*t*_ denotes standard medical conditions (including medical facilities, medical technology etc.), mortality and infection rates during Covid-19, respectively. $C_{t}^{O}$ denotes outdoor consumption. Therefore, ${\left(J^{\psi_{t}}\right)}_{t}C_{t}^{O}+C_{t}^{I}=C_{t}$.

${\left(J^{\psi_{t}}\right)}_{t}=(0,1)$ represents outdoor activities (including travel) which are dummy variables, that is when ***ψ***_*t*_ = 0, the government does not impose a travel ban and representative families are free to exercise outdoors; when ***ψ***_*t*_ = 1, the government imposes a travel ban and representative families follow the ban. ${\left(J^{\psi_{t}}\right)}_{t}C_{t}^{O}$ represents whether the government practiced (did not practice) a policy of restraint, if ${\left(J^{\psi_{t}}\right)}_{t}=1({\left(J^{\psi_{t}}\right)}_{t}=0).$ *S*_*t*_ denotes household state, that is, $S_{t}=\{E_{t},M_{t},F_{t},G_{t}^{h}\psi_{t},C_{t},\epsilon_{t}^{J}\}$. *ϑ* is parameter. $\epsilon_{t}^{J}$ is unobserved utility received from ***J***.

Let $C_{t}+H(E_{t},M_{t},F_{t},G_{t}^{h}\psi_{t};\vartheta )+\epsilon_{t}^{J}=\bar{U}(G_{t}^{h}\psi_{t})$, Using [23] equation, the dynamic programming can be expressed as follows:

$$V(S_{t},\epsilon_{t}^{J}|\theta_{t},\psi_{t})=\bar{U}(G_{t}^{h}\psi_{t})+\epsilon_{t}^{J}+\beta [\sum_{\psi_{t+1}}\pi (\psi_{t+1}|S_{t+1})\sum_{S_{t+1}}P_{S_{t}S_{t+1}}^{\psi_{t}}V(S_{t+1},\epsilon_{t+1}^{J}|\theta_{t+1},\psi_{t+1})]$$ (3.1.3)

Where *β* is the discount rate. $\pi (\psi_{t+1}|S_{t+1})$ denotes environment transition probability, that is, actions $\psi_{t+1}\in \Psi =\{\psi_{t},\psi_{t+1},\ldots ,\psi_{t+n}\}$ taken by representative families to maintain their state *S*_*t*+1_. $P_{S_{t}S_{t+1}}^{\psi_{t}}$ is the state transition probability, that is, the probability that the state of a representative household transitions from *S*_*t*_ to *S*_*t*+1_ after acting ***ψ***_*t*_ in period *t*. To simplify the model, we assume the probability that the state of a representative household transitions from *S*_*t*_ to *S*_*t*+1_ is equal to 1, and since representative families are based on perfectly rational assumptions, they choose the optimal action (***ψ***_*t*_)*. Therefore, the optimal Bellman equation can be re-expressed as follows:

$$V(S_{t},\epsilon_{t}^{J}|\theta_{t},\psi_{t})=\bar{U}(G_{t}^{h}\psi_{t})+\epsilon_{t}^{J}+\beta V(S_{t+1},\epsilon_{t+1}^{J}|\theta_{t+1},\psi_{t+1})⟹V(S_{t+1},\epsilon_{t+1}^{J}|\theta_{t+1},\psi_{t+1})=E(\underset{\psi_{t+1}}{\max}V(S_{t+1},\epsilon_{t+1}^{J}|\theta_{t+1},\psi_{t+1}))$$

We also assume that there is a time lag from the time a government announces the restriction policies to the time the government implements them. Therefore, there may be dynamic inconsistencies. The representative family only knows the probability density function of the restriction policy *f*(*ψ*), *ψ* which follows logistic distribution. Therefore, Eq (3) can be re-written under uncertainty:

$$E(V(S_{t},\epsilon_{t}^{J}|\theta_{t},\psi_{t}))=\int_{R_{+}}V(S_{t},\epsilon_{t}^{J}|\theta_{t},\psi_{t})f(\psi_{t})d\psi_{t}$$

We use backward recursion to solve the Eq (3). Thus, the initial value can be expressed as follows:

$$V(S_{0},\epsilon_{0}^{J}|\theta_{0},\psi_{0})=\beta \left[\sum_{\psi_{0}}\pi (\psi_{1}|S_{1})\sum_{S_{1}}P_{S_{0}S_{1}}^{\psi_{0}}V\left(S_{1},\epsilon_{1}^{J}|\theta_{1},\psi_{1}\right)\right]$$

To sum up, the initial value problem can be written as follows:

$$\left\{ \begin{array}{c} V(S_{t},\epsilon_{t}^{J}|\theta_{t},\psi_{t})=\bar{U}(G_{t}^{h}\psi_{t})+\underset{\psi_{t+1}}{\max}\left\{\beta \int_{R_{+}}V(S_{t+1},\epsilon_{t+1}^{J}|\theta_{t+1},\psi_{t+1})f(\psi_{t+1})d\psi_{t+1}\right\}\\ s.t.V(S_{0},\epsilon_{0}^{J}|\theta_{0},\psi_{0})=\beta [\sum_{\psi_{0}}\pi (\psi_{1}|S_{1})\sum_{S_{1}}P_{S_{0}S_{1}}^{\psi_{0}}V(S_{1},\epsilon_{1}^{J}|\theta_{1},\psi_{1})] \end{array}\right.$$ (3.1.4)

### 3.2 Data

Since panel data contains more information and can minimize estimation bias [24], we chose panel data analysis based on eight countries: China, the United States, Canada, Australia, France, Italy, Japan, and South Korea. These countries are selected because they have been greatly affected by COVID-19 and/or have complete relevant data. Other countries that are heavily affected by COVID-19, such as the United Kingdom, Brazil, and India, have not been included in this paper due to incomplete statistical data. Our aggregate sample data are monthly.

In fact, we face the problem of insufficient control variables due to lack of data. According to the conclusion of the economic model above, our control variables should have included age, gender, outdoor consumption per month, indoor consumption per month, total number of hospitals per month, ICU beds per month, average education level of medical staff, and happiness index of citizens. Therefore, for those variables that lack of data, we can only substitute other variables for observations. We use a country’s economic growth rate to indirectly observe a country’s health environment, including total number of hospitals per month, ICU beds per month, average education level of medical staff. We can indirectly observe the health environment of a country through its economic growth rate. It is true that the health environment observed through economic growth rates is not very accurate. However, since we use the monthly data from 2020, which is micro data, the statistical imperfections of the healthcare environment in various countries leads us to use other control variables to replace the original control variables. We reviewed many databases online, and we originally intended to replace the growth rate of the economy with better health care expenditure as a control variable for the health care environment. However, we found that countries’ health expenditure was only counted up to 2018 or 2019. For the above reasons, we chose the economic growth rate as a surrogate variable for the medical environment. The better a country’s economic development, the better its infrastructure and the more it invests in people’s livelihoods. We will further explain the econometric variables in section 4.

The original sample data of the dependent variable (the number of confirmed cases) and the independent variable (the government response) are collected from the Stringency Index for Government Response during COVID-19 (from 2020-01-01 to 2021-02-30) of the University of Oxford [25], which are collected in the form of daily statistics (Source: https://ourworldindata.org/grapher/covid-stringency-index). [25] launched the Oxford COVID-19 Government Response Tracking System (OxCGRT), which provides a systematic way to track national and local jurisdictions’ response to COVID-19 over time. They combined the data into a series of novel indices that combined various government responses. These indices are used to describe changes in these responses, explore whether they affect infection rates, and determine correlations between different degrees of response. Our raw data of control variables (GDP growth rate and inflation rate) and instrument variable (Government Budget) have been collected from the Take-Profit Organization website (Source: https://take-profit.org/en/). We used the quadratic interpolation to convert frequency for these data from daily to monthly.

### 3.3 Econometric approach

Our data belong to large “*T*” (Time) and small “*N*” (Cross-section). Thus, we need to carry out a unit root test, co-integration test and causality test on the time series of variables to ensure their stationarity, their long-term equilibrium relationship and their statistical causality. The following unit root tests will be used to underpin our paper: [26–28], augmented Dickey–Fuller (ADF) and Phillips-Perron (PP). We will use the covariance test of [29] and [30] to test whether there is a long-term equilibrium relationship between our variables.

We briefly introduce two methods of unit root test extended by the ADF test. [26] proposed the LLC test method. The LLC test adopts the ADF test form as follows:

$$\Delta y_{it}=\eta y_{it-1}+\sum_{j=1}^{p_{i}}\beta_{ij}\Delta y_{it-j}+x_{it}^{\prime }\delta +u_{it},\;i=1,2,\ldots ,N\;t=1,2,\ldots ,T$$

Where *η* = *ρ*−1, *p*_*i*_ denotes the lag order of the *i*th cross-section. The null hypothesis of the LLC test is that each cross-section sequence in panel data has the same unit root, and the alternative hypothesis is that each cross-section sequence has no unit root, that is, *H*_0_: *η* = 0, *H*_1_: *η*<0. The [27] test is similar to the LLC test. Its null hypothesis denotes that each cross-section sequence in panel data has a unit root, and proxy variables of Δ*y*_*it*_ and *y*_*it*−1_ are used to estimate parameters *η*. However, the form of proxy variables in the Breitung test is different from that in LLC test. The Breitung test first eliminates the influence of dynamic item Δ*y*_*it*−1_ from Δ*y*_*it*_ and *y*_*it*−1_, then standardizes and obtains the corresponding proxy variables through regression, and finally uses the proxy variables to make regression $\Delta y_{it}^{*}=\eta y_{it-1}^{*}+\varepsilon_{it}$, and estimate parameters *η*.

[28] further extended the LLC test. They first performed the following unit root test on each cross-section member:

$$\Delta y_{it}=\eta_{i}y_{it-1}+\sum_{j=1}^{p_{i}}\beta_{ij}\Delta y_{it-j}+x_{it}^{\prime }\delta +\varepsilon_{it},\;i=1,2,\ldots ,N\;t=1,2,\ldots ,T$$

Therefore, the null hypothesis of the Im-Pesaran-Shin test is *H*_0_: *η*_*i*_ = 0, for all *i*, the alternative hypothesis is $H_{1}:\left\{ \begin{array}{c} \eta_{i}=0,\;for\;i=1,2,\ldots ,N_{1}\\ \eta_{i}<0,\;for\;i=N_{1}+1,N_{1}+2,\ldots ,N \end{array}\right.$. After the unit root test for each cross-section, they use the *t* statistics: $t_{iT_{i}}(p_{i})$ of a single cross-section to construct whether the whole panel data has the *t* statistics: ${\bar{t}}_{NT}=\frac{\left[\sum_{i=1}^{N}t_{iT_{i}}(p_{i})\right]}{N}$ of the unit root. If the cross section contains the lag term, it provides a statistic of asymptotic normal distribution using $W_{{\bar{t}}_{NT}}=\frac{\sqrt{N}({\bar{t}}_{NT}-N^{-1}\sum_{i=1}^{N}E(t_{iT_{i}}(p_{i})))}{\sqrt{N^{-1}\sum_{i=1}^{N}var(t_{iT_{i}}(p_{i}))}}\to N(0,1)$.

[29] proposed the co-integration test method of panel data based on the two-step method of Engle and Granger. Based on the regression residual of the co-integration equation, this method constructed 7 statistics to test the co-integration relationship among panel variables. The null hypothesis of the Pedroni test is that there is no co-integration relationship between panel variables. The Pedroni test assumes that cross-section individuals are independent of each other and the error process is stable, and its asymptotic covariance matrix is $\Omega_{i}=\underset{T\to \infty }{\lim}E[T^{-1}(\sum_{t=1}^{T}w_{it})(\sum_{t=1}^{T}w_{it}^{\prime })]=\Omega_{i}^{0}+\Gamma_{i}+\Gamma_{i}^{\prime }$, where $\Omega_{i}^{0}$ denotes covariance, ***Γ***_*i*_ denotes the weighted sum of auto-covariance. Pedroni used the following assistant regression: ${\hat{u}}_{it}=\rho_{i}{\hat{u}}_{it-1}+v_{it},\;i=1,2,\ldots ,N$ to test $y_{it}=\alpha_{i}+\delta_{i}t+x_{it}^{\prime }\beta_{i}+u_{it}$ to obtain whether the residual sequence is stable. In the stationarity test of residual errors, the specific null hypothesis and alternative hypothesis used by Pedroni can be divided into two situations: <1. $H_{0}:\;\rho_{i}=1,\;H_{1}:\;(\rho_{i}=\rho )<1$. <2. $H_{0}:\;\rho_{i}=1,\;H_{1}:\;\rho_{i}<1$.

[30] uses the same basic method as the Pedroni test. However, the biggest difference between them is that in the first stage of the Kao test, the regression equation is set as each cross-section individual has different intercept terms and the same coefficient, and all trend coefficients are set as 0.

One of the advantages of panel data is that it enables researchers to better understand the dynamic adjustment process. The dynamic panel data has the common feature that the regression variables contain delayed explained variables. Therefore, based on the dynamic programming (3.1.4), our regression model adopts the differential generalized moment estimation method proposed by [31] and the orthogonal generalized moment estimation method proposed by [32]. Our form of regression equation can be expressed as follows:

$$y_{it}=\sum_{h=1}^{p}\phi_{h}y_{it-h}+\sum_{k=1}^{K}\beta_{k}x_{kit}+\upsilon_{it},\;i=1,2,\ldots ,N\;t=1,2,\ldots ,T$$ (3.3.1)

Where *υ*_*it*_ = *α*_*i*_+*u*_*it*_.

Based on the long-term equilibrium relationship of the variables, we first estimate the coefficients of the variables by the ordinary least square method, and then estimate the coefficients of the variables by the dynamic generalized moment estimation method. In the estimation of panel data, the sample data uses contains information from three dimensions of cross-section, time, and variables. If the model form is set incorrectly, the estimation result will deviate from the economic reality. Therefore, to avoid model setting bias, we first check which panel data model form the sample data needs to use. There are three different models of panel data: <1. Constant coefficient model: $y_{i}=\alpha e+x_{i}\beta +u_{i},\;i=1,2,\ldots ,N$; <2. Intercept change model: $y_{i}=\alpha_{i}e+x_{i}\beta +u_{i},\;i=1,2,\ldots ,N$; <3. Varying-coefficient model: $y_{i}=\alpha_{i}e+x_{i}\beta_{i}+u_{i},\;i=1,2,\ldots ,N$. We used an analysis of covariance test to determine the correct panel model. The covariance test tests two hypotheses: $H_{1}:\;\beta_{1}=\beta_{2}=\cdots =\beta_{N};\;H_{2}:\;\left\{ \begin{array}{c} \alpha_{1}=\alpha_{2}=\cdots =\alpha_{N}\\ \beta_{1}=\beta_{2}=\cdots =\beta_{N} \end{array}\right.$. If the hypothesis *H*_2_ is accepted, then we choose the constant coefficient model. If we reject hypothesis *H*_2_, we need to further test hypothesis *H*_1_. If hypothesis *H*_1_ is accepted, then we choose the variable intercept model. If we reject the hypothesis *H*_1_, we choose the varying-coefficient model. We calculate the sum squares of residuals for the three different models $S_{1},\;S_{2},\;S_{3}\;(S_{1}=\sum_{i=1}^{N}RSS_{i},\;S_{2}=W_{yy}-W_{xy}^{\prime }W_{xx}^{-1}W_{xy},\;S_{3}=T_{yy}-T_{xy}^{\prime }T_{xx}^{-1}T_{xy}$, where $W_{xx,i}=\sum_{t=1}^{T}(x_{it}-\bar{x_{i}}){\left(x_{it}-\bar{x_{i}}\right)}^{\prime },$ $W_{xy,i}=\sum_{t=1}^{T}{\left(x_{it}-\bar{x_{i}}\right)}^{\prime }(y_{it}-\bar{y_{i}}),$ $W_{yy,i}={\sum_{t=1}^{T}\left(y_{it}-\bar{y_{i}}\right)}^{2},$ $W_{xx}=\sum_{i=1}^{N}W_{xx,i},$ $W_{xy}=\sum_{i=1}^{N}W_{xy,i},$ $W_{yy}=\sum_{i=1}^{N}W_{yy},$ $T_{xx}=\sum_{i=1}^{N}\sum_{t=1}^{T}(x_{it}-\bar{x_{i}}){\left(x_{it}-\bar{x_{i}}\right)}^{\prime },$ $T_{xy}=\sum_{i=1}^{N}\sum_{t=1}^{T}{\left(x_{it}-\bar{x_{i}}\right)}^{\prime }(y_{it}-\bar{y_{i}}),$ $T_{yy}=\sum_{i=1}^{N}{\sum_{t=1}^{T}\left(y_{it}-\bar{y_{i}}\right)}^{2},$ $\bar{x}=\frac{1}{NT}\sum_{i=1}^{N}\sum_{t=1}^{T}x_{it},$ $\bar{y}=\frac{1}{NT}\sum_{i=1}^{N}\sum_{t=1}^{T}y_{it}$). and under hypothesis *H*_2_, the test statistic *F*_2_ is the *F* distribution of corresponding degrees of freedom, namely:

$$F_{2}=\frac{\left(S_{3}-S_{1})/[\left(N-1)(k+1\right)\right]}{S_{1}/\left[NT-N(k+1)\right]}∼F[\left(N-1)(k+1),N(T-k-1\right)]$$

If the value of the calculated statistic *F*_2_ is not less than the corresponding critical value under the given confidence, then the hypothesis *H*_2_ need to be rejected and the hypothesis *H*_1_ needs to continue to be tested. Instead, we choose the constant coefficient model. Under hypothesis *H*_1_, the test statistic *F*_1_ is the *F* distribution of corresponding degrees of freedom, namely:

$$F_{1}=\frac{\left(S_{2}-S_{1})/[(N-1)k\right]}{S_{1}/\left[NT-N(k+1)\right]}∼F[\left(N-1)k,N(T-k-1\right)]$$

If the value of the calculated statistic *F*_1_ is not less than the corresponding critical value under the given confidence, then the hypothesis *H*_1_ needs to be rejected and we choose the varying-coefficient model. Instead, we choose the Intercept change model.

We get 46.3763, 75.9119, and 75.5645 for *S*_1_, *S*_2_, and *S*_3_, respectively. *N* = 8, *k* = 4 and *T* = 11. Thus, *F*_2_ = 0.8632<*F*[35,48]. Therefore, we choose the constant coefficient model. In order to eliminate the heteroscedasticity of residuals and sequence autocorrelation, the cross-section SUR weighted method is used to estimate the parameters in OLS and TSLS. Cross-section SUR weighting is also used to estimate the covariance of the coefficients.

## 4. Empirical analysis and implications

We first state the variables: in dynamic programming (3.1.3), $V(S_{t},\epsilon_{t}^{J}|\theta_{t},\psi_{t})$, the dependent variable denotes the heath state of citizens. Since it is a latent variable, we choose the Case fatality rate (*CFR*) in COVID-19 as the variable to measure $V(S_{t},\epsilon_{t}^{J}|\theta_{t},\psi_{t})$. Case fatality rate is the ratio of the number of people who die from a disease in a given period to the total number of people diagnosed with the disease [33]. The lower the case fatality rate, the less serious the pandemic. Our independent variable is *GOV*_*R*, which represents the growth rate of government response. According to the report of government response in COVID-19 [25], the larger the government response, the higher the value. We observe *C*_*t*_ by control variable *INF*, which represents the inflation rate and indirectly reflects the consumption state of a country. We choose GDP growth rate (*GDP*_*G*) as one of the control variables, which can indirectly reflect the medical environment of a country and the possible medical expenditure of the government in response to emergency public health. In fact, we face the problem of insufficient control variables due to lack of data. Again, according to the conclusion of the economic model above, our control variables include age, gender, outdoor consumption per month, indoor consumption per month, total number of hospitals per month, ICU beds per month, average education level of medical staff, and happiness index of citizens. Therefore, for those variables that lack of data, we can only substitute other variables for observations. Table 1 shows the variable description.

**Table 1:** Variable description.

Variable	Description
*CFR*	COVID-19 case fatality rate
*INF*	Inflation rate
*GOV*_*R*	Growth rate of government response
*GDP*_*G*	GDP growth rate
*CFR*(−1)	COVID-19 case fatality rate of lag phase 1

According to programming (3.1.4) and Eq (3.3.1), we consider the cyclical relationship between the growth rate of government response and the case fatality rate in the absence of a vaccine, namely, the volatility and periodicity of pandemic. Thus, we assume $H(E_{t},M_{t},F_{t},G_{t}^{h}\psi ;\vartheta )$ is $\frac{1}{GOV\_R}\cos\left(GOV\_R\right),\bar{U}(G_{t}^{h}\psi_{t})=\alpha_{1}\frac{1}{GOV\_R}\cos\left(GOV\_R\right)+\alpha_{2}INF+\alpha_{3}GDP\_G$. Therefore, the form of our regression equation is set as follows:

$$CFR=\alpha_{2}INF+\sum_{k=1}^{K}\alpha_{1k}\frac{1}{GOV\_R}\cos\left(GOV\_R\right)+\alpha_{3}GDP\_G+\sum_{h=1}^{p}\beta_{h}({CON\_C)}_{it-h}+\upsilon_{it},\;i=1,2,\ldots ,N\;t=1,2,\ldots ,T$$ (4.0.1)

Table 2 shows the mean standard deviations of the sample population and each section. Overall, the average case fatality rate is stable at 3%, up from the previous period. Governments responded by average growth at 0.6%, showing that the pandemic control around the world had some success. In terms of individual cross-section, Japan has the lowest average (−0.01) and China has the lowest standard deviation (0.00) of the case fatality rate, indicating that the pandemic is effectively controlled (with lower outbreak risk) in China. The highest average growth rate of case fatality rate is in Canada. Standard deviation in France is the highest, showing that the pandemic is not well controlled in France. From the independent variable *GOV*_*R*, the average growth rate of government response is the lowest in China and Australia, and both of their standard deviations are small. The lowest standard deviation and mean denote that the anti-pandemic response of China has entered a convergence state. During COVID-19, the country with the highest average GDP growth rate was Canada while the high standard deviation indicates that Canada’s GDP growth was not stable. Average GDP growth in most countries is negative, which may indirectly indicate the impact of COVID-19 on economic growth.

**Table 2:** Descriptive statistics of data.

		*CFR*	*INF*	*GOV_R*	*GDP_G*	*CFR*(−1)
All sample countries	Mean	0.003	0.077	0.006	0.012	0.002
	Std. Dev.	0.018	0.607	0.016	0.078	0.019
China	Mean	0.001	-0.190	0.001	0.020	0.003
	Std. Dev.	0.004	0.682	0.005	0.048	0.007
United States	Mean	0.003	0.060	0.011	-0.044	0.003
	Std. Dev.	0.018	0.435	0.023	0.036	0.018
Canada	Mean	0.005	0.040	0.008	0.098	0.006
	Std. Dev.	0.021	0.462	0.025	0.298	0.021
Australia	Mean	0.002	0.350	0.004	-0.034	0.002
	Std. Dev.	0.024	1.301	0.012	0.030	0.024
France	Mean	0.004	0.050	0.006	-0.090	0.000
	Std. Dev.	0.026	0.237	0.013	0.066	0.030
Italy	Mean	0.002	-0.020	0.005	-0.098	0.000
	Std. Dev.	0.023	0.294	0.012	0.058	0.024
Japan	Mean	-0.001	-0.070	0.005	-0.057	-0.001
	Std. Dev.	0.010	0.164	0.014	0.039	0.010
South Korea	Mean	0.004	0.400	0.006	-0.017	0.004
	Std. Dev.	0.016	0.435	0.015	0.017	0.016

### 4.1 Panel unit root test

As stated before, in order to make the time series stable, we do the stationarity test for the time series of various variables. We use four unit root tests: the [26] test, [27] test, [28] test, augmented Dickey–Fuller (ADF) test and Phillips-Perron (PP) test. Table 1 in S1 Appendix presents the level and first order difference test results of unit roots of each variable under four different methods. Table 3 shows that all variables are single integer at levels, that is *I*(0).

**Table 3:** Panel co-integration results.

Pedroni (1999, 2004) residual co-integration test.			
	within-dimension	Statistics	P-value
Panel v-Statistic		-1.941	0.97
Panel rho-Statistic		2.347	0.99
Panel PP-Statistic		-3.672***	0.00
Panel ADF-Statistic		-2.592***	0.01
	between-dimension	Statistics	P-value
Group rho-Statistic		3.871	1.000
Group PP-Statistic		-4.485***	0.000
Group ADF-Statistic		-2.827***	0.002
Kao (1999) residual cointegration test			
		Statistics	P-value
ADF		-9.214***	0.00

Notes

*** denotes significance level at 1%. The null hypothesis of co-integration is there is no co-integration relationship among variables; the alternative hypothesis is there is co-integration relationship among variables.

### 4.2 Panel co-integration test

Using the co-integration test method of [29] and [30], we conduct the co-integration test for the long-term relationship between variables. Kao test results show that the test passed the co-integration test. Pedroni test results show that half of the statistics pass the co-integration test. After comprehensive analysis, we believe that there is a long-term equilibrium relationship between the variables.

### 4.3 Regression results

Table 4 shows the regression results under four regression methods. Empirically, there is a two-way causality between the growth rate of government response and the case fatality rate, that is, an increase (decrease) in the growth rate of government response will lead to a decrease (increase) in the case fatality rate. Thus, two-way causality creates possible endogenous problems. To solve the endogenous problem, we used TSLS and a dynamic GMM method to re-estimate the parameters. The results show that the parameters estimated by TSLS and dynamic GMM method are basically consistent with the parameters estimated by the OLS method, which verifies the effectiveness of our parameter estimation.

**Table 4:** Regression results of entire panel.

Methodology and Variables	parameters	OLS	TSLS	GMM-Difference	GMM-Orthogonal
(1/*GOV*_*R*)⋅cos(*GOV*_*R*)	*α*_1_	0.0792*** (3.92)	0.1203*** (1374.72)	0.0842*** (3.91)	0.1580** (2.50)
*INF*	*α*_2_	-0.0013** (-2.15)	-0.0018*** (-1000.13)	-0.0021*** (-4.29)	0.0001 (0.09)
*GDP*_*G*	*α*_3_	-0.0332*** (-6.91)	-0.0286*** (951.79)	-0.0334*** (-7.72)	-0.0415* (-1.74)
*CFR*(−1)	*β*	0.0344 (0.65)	0.0607*** (116.51)	0.2328*** (25.28)	0.2615*** (5.15)
Adjusted *R*^2^		0.453	0.99		
*D*. *W*.		1.951	1.867		
Arellano-Bond Serial Correlation Test *AR*(1)				-1.7010* (0.087)	
Arellano-Bond Serial Correlation Test *AR*(2)				-1.4861 (0.137)	
*J* Statistics				2.866	2.614
*P* value of *J* Statistics			0.267	0.580	0.624

Notes

***, ** and * denote significance level at 1%, 5% and 10%, respectively.

We choose government budget (*GOV*_*E*) and economic support (*ECO*_*S*) during COVID-19 as instrumental variables. The selection of instrumental variables needs to be highly correlated with endogenous independent variables and independent of random disturbance. The main government response is from the government budget. Economic support during the pandemic is also the most effective way to control the pandemic. Therefore, both government budget and economic support are intermediate variables, which are highly correlated with independent variables and independent of the disturbance term.

It is not difficult to find that our independent variable is significant at the 1% and 5% confidence level. Under OLS, TSLS and dynamic GMM methods, *D*.*W*.∈[1.59,2.31] (In the TSLS, the initial *D*.*W*. is 0.9198, indicating that the model has positive first-order autocorrelation. Therefore, after we added AR (1), *D*.*W*. becomes 1.867, indicating that there is no first-order sequence autocorrelation in the model) proves there is no first order sequence auto-correlation. The *P* value of the Hansen *J* statistic shows that the null hypothesis of [34] test cannot be rejected. Therefore, the moment condition of over-recognition is valid, that is, the instrumental variables selected by our model are valid. The first-order statistics of the Arellano-Bond test for the differential GMM model are significant under the 10% level. The second-order statistics are not significant, which indicates that the model does not have second-order sequence auto-correlation. It shows that our model has been set up correctly.

**Fig 1:**
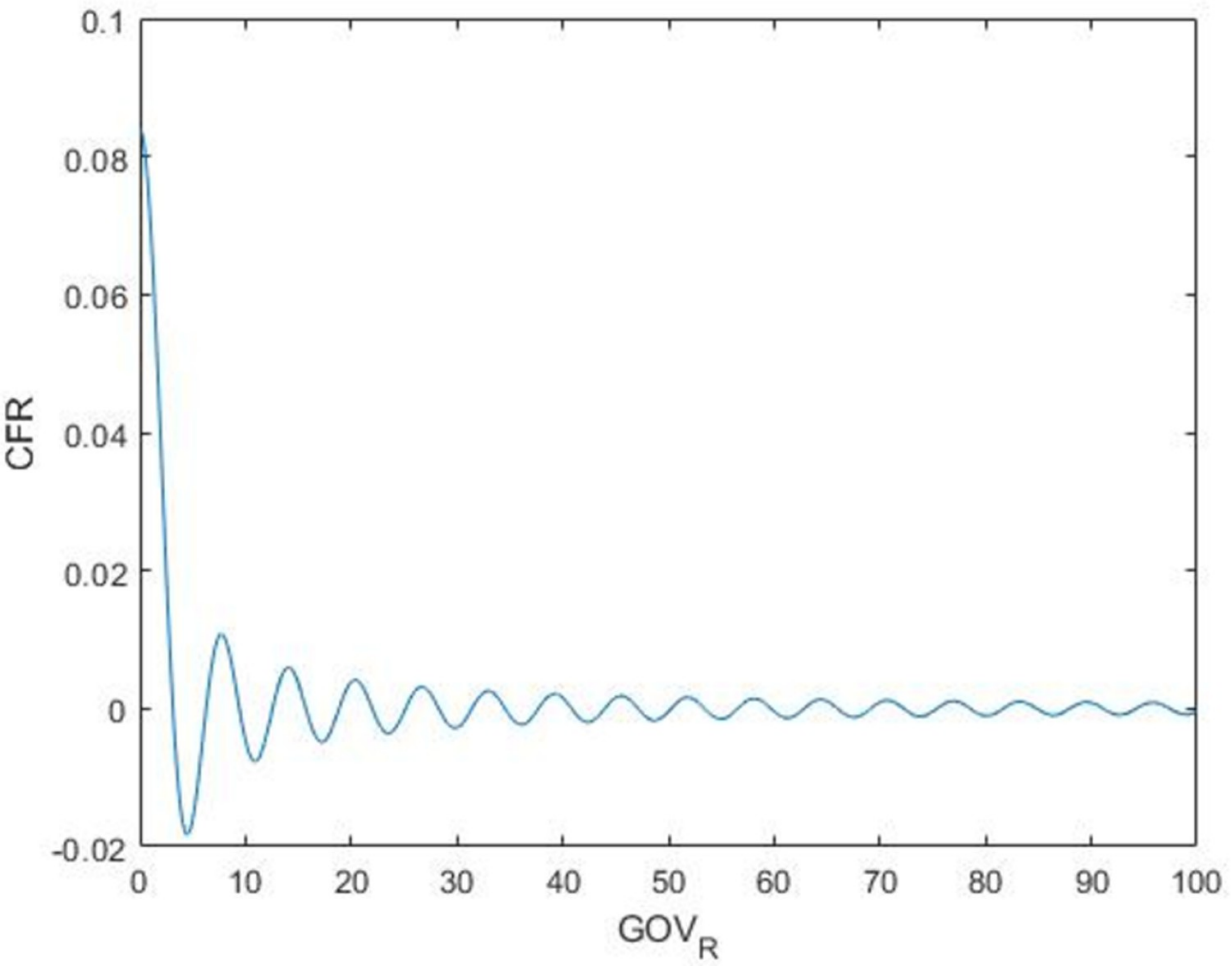
Relationship between *CFR* and *GOV_R*.

The estimation results of the four methods show that the parameters *α*_1_, and *β* are significantly positive. The results show that the outbreak of COVID-19 has inertia characteristics. The case fatality rate of the previous period will significantly affect the case fatality rate of the current period. It means that if the government had not taken timely measures to control the COVID-19 outbreak in the previous period, then the government would need to take more measures for the current period to control COVID-19. There is a cyclical relationship between the growth rate of government response and the case fatality rate of COVID-19 (see Fig 1).

The *CFR* fluctuates as the government’s response increases. $\underset{{\left(\mathrm{GOV}\_R\right)}_{t}\to 0^{+}}{\lim}{\left(CFR\right)}_{t}=0$. When COVID-19 does not occur at the beginning, the government response is 0, and the case fatality rate is 0. Looking back, by the time the government paid attention to COVID-19, the pandemic had already reached a certain acuteness. The government increased the level of response, and the pandemic began to be effectively controlled. Case fatality rate dropped rapidly to the local minimum value. It can be seen that in addition to government response, other factors will also cause the fluctuation of the Case fatality rate. After reaching the local minimum point, the Case fatality rate rises to the local maximum point again, and then decreases to the second local minimum point until it converges to near 0. Therefore, the fluctuation process of the Case fatality rate decreases and finally stabilizes around 0. From the influence of the growth rate of government response on the case fatality rate, it is not difficult to find that the growth rate of government response has a cyclical change on the case fatality rate. In response to the increasing growth rate of the government, the case fatality rate first increases, and then decreases to the minimum point after reaching the maximum point. After reaching the minimum point, the case fatality rate goes up again. Finally, there is a cyclical fluctuation in the rate of case fatality rate, and it will converge to 0. There is a negative correlation between economic growth rate and inflation rate on case fatality rate.

Our regression results are consistent with those presented by the statistical description. The government response has been the most effective way of controlling the scale of the COVID-19 outbreak. China first used strict restrictive policies to control the development of COVID-19 in China, and finally succeeded in controlling the development of the pandemic [35]. Since then, China has resumed production and daily activities, and the economy has gradually begun to recover [36]. This is in line with our suggestion: in the stage of rising pandemic scale, the government should aim to control the pandemic and improve economic development; in the declining stage of the pandemic development, the government should take economic development as the goal and make the government response. Australia is one of the most successful countries in controlling COVID-19. The Australian government adopted a rigorous government response in the early stages of the outbreak and achieved effective results [6]. The positive impact of an active government response in controlling the development of COVID-19 is well illustrated by the cases of China and Australia. Strict restrictions are the most important part of the government’s response. Strict restrictions, including social distance restrictions, mandatory mask wearing, travel bans, etc., are positive government responses. According to our findings, if the government maintains a positive response, based on the cyclical relationship between the government response and the COVID-19 fatality rate, the government will have fewer costs and be able to contain the epidemic for some time. Japan and South Korea also adopted relatively strict government response policies after COVID-19 occurred and achieved the expected results [37].

### 4.4 Discussion and implication

Some actual evidence supports our regression results that the growth rate of the government response has a cyclical effect on the case fatality rate. The first case of community transmission in Canada was confirmed on 5 March 2020. The Canadian government immediately declared a state of emergency [38]. Canadian provinces and territories have, to varying degrees, implemented closures of schools and day care facilities, bans on gatherings, closures of non-essential businesses and restrictions on access. The Canadian government at that time required all travellers returning to the country to self-quarantine for 14 days. After that, active cases in Canada continued to decline until late summer and early winter. In September 2020, cases rebounded in all Canadian provinces and territories [39]. On 23 September 2020, the Canadian government announced that Canada was experiencing a second wave of the epidemic [40]. As cases have increased, the government has again imposed new restrictions, including lockdowns in different areas. In December 2020 and January 2021, cases, hospitalizations, and deaths in Canada spiked around the Christmas and holiday seasons. Therefore, the Canadian government imposed strict restrictions (such as lockdowns and curfews) again across the country. These closures led to a steady decline in active cases [41]. Following the third and fourth wave, which occurred between March and October 2021, the Canadian local government reinstated the travel ban. At the time of the fourth wave, it was also referred to as an "unvaccinated pandemic" because of Canada’s high vaccination rates. Similarly, Canadian provincial and territorial governments have reimposed restrictions around travel and quarantine.

France has already experienced three waves of the virus. On 12 March 2020, in the first wave of the outbreak, the French government announced the closure of all schools and universities, a ban on gatherings of more than 100 people, excluding public transport, and the closure of all non-essential public places including restaurants, cafes, cinemas and nightclubs [42]. Since then, the French government gradually lifted the blockade [43]. From August 2020, the rate of infection increased, with France recording 26,896 new infections in Europe in a 24-hour period on 10 October 2020. This increase led France to enter a second nationwide lockdown on 28 October 2020. The French government announced its third nationwide lockdown from April 3, 2021. The restrictions included the closure of non-essential stores, the suspension of schools, a ban on domestic travel and a nationwide curfew from 7pm to 6am.

Italy experienced the first wave of the epidemic from February 2020. The government suspended all flights and declared a state of emergency. The Italian government closed all non-core businesses and industries and restricted the movement of citizens [44]. Restrictive measures had had an initial positive effect [45]. By May 2020, many restrictions were gradually eased and freedom of movement between Italian regions and other European countries was restored [46, 47]. In October 2020, As Italy began to experience the impact of the second wave of the epidemic, the government introduced further actions and restrictions on social life. All hospital facilities had been upgraded and expanded, with more beds and intensive care units than in March 2020. Tracking applications, monitoring systems, and prediction systems were used to understand the progress of the outbreak. The Italian government forced the closure of gyms, swimming pools, theatres, and cinemas, as well as bars and restaurants by 6pm [48].

So far, there have been four outbreaks in Japan. The first wave occurred in January 2020, when COVID-19 was transmitted by a passenger from China [49]. A large cluster of infections was detected in March. The source of the second wave of transmission was a variant of the European virus, which Japanese experts believe was transmitted by travelers from Europe between March 11, 2020, and March 23, 2020. The third wave began in August 2020, when the number of confirmed cases in Japan reached 230,000 and the death toll surpassed 100,000. The fourth wave occurred in April 2021, and more than 4,000 new cases were confirmed, leading the Japanese government to declare a fourth wave.

Former U.S. President Donald Trump adopted a negative prevention and control policy, which led to the continuous expansion of the COVID-19 in the United States, and seriously affected production activities and economic development [50]. The United States has experienced at least three waves of the pandemic. The first wave was from March to July. The number of people diagnosed in the United States reached 50,000 during this period. The second wave appeared between July and October. The number of people diagnosed in the United States reached 100,000 during this period. The third wave began in October, with the number of confirmed cases rising rapidly to more than 200,000.

On November 5^th^, Britain had to go into a second national lockdown because of a rise in cases and hospitalizations. After the blockade ended on 2 December, the number of cases began to rise again, with more than 70,000 deaths from COVID-19 as of 11 December 2020.On 4 January 2021, British Prime Minister Boris Johnson addressed the nation and announced the third blockade. Therefore, the UK was put into lockdown, more contagious variant of COVID-19 spread across the UK, causing a rapid increase in cases and deaths [51]. It is now reopening and coming out of lockdown.

**Fig 2:**
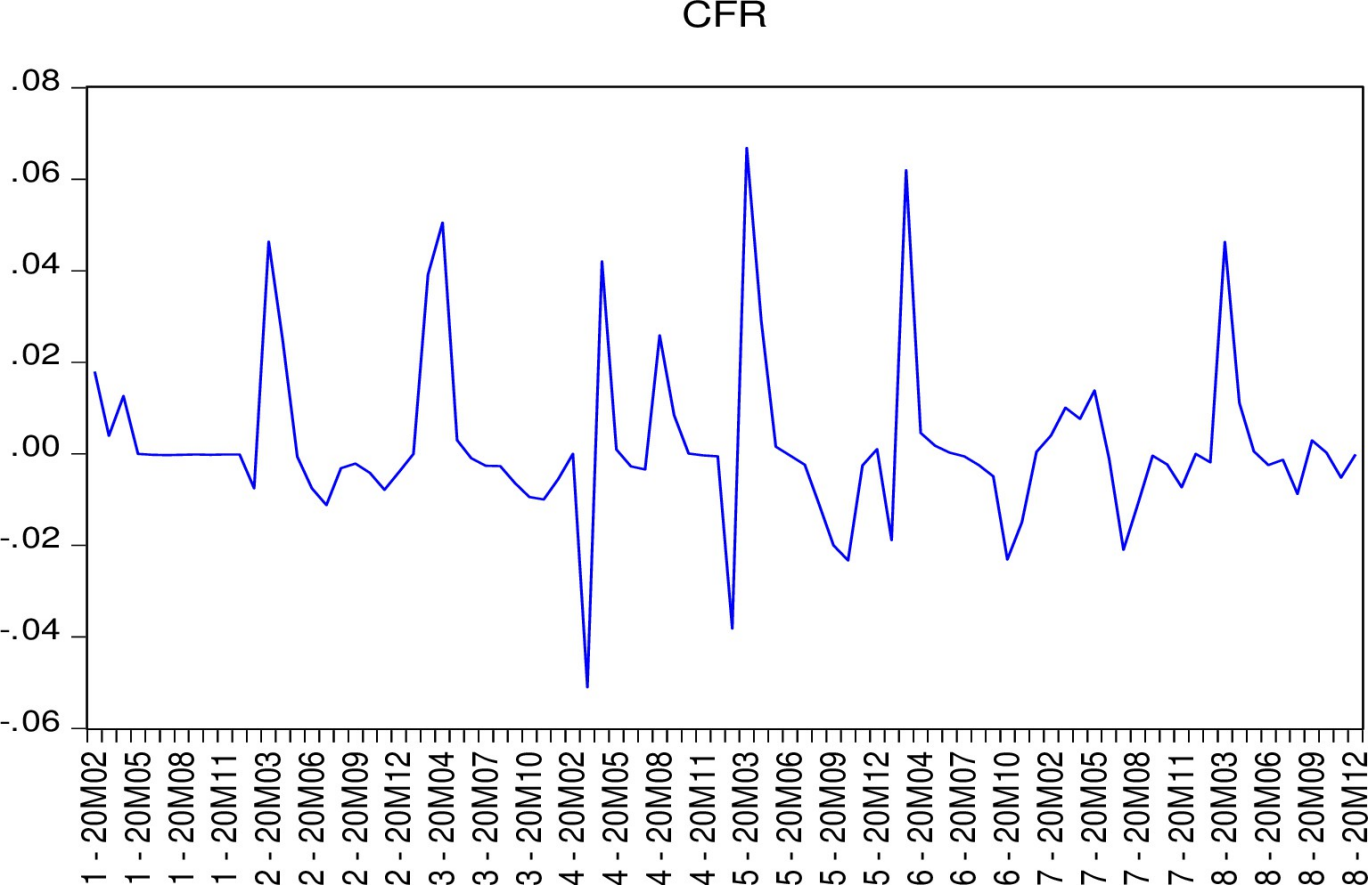
Case fatality real trend from 8 countries.

Fig 2 shows the actual case fatality rate trends for the eight countries. It is not difficult to find in these eight countries, the *CFR* has a significant fluctuation. This confirms the validity of our empirical results. We believe that multiple factors are responsible for the cyclical fluctuation of the pandemic. Firstly, the COVID-19 virus has mutated. The mutation of the virus is difficult to control. The virus mutates to increase the rate of infection or death. UK COVID-19 virus mutation was first detected in samples collected last month in Kent during the UK COVID-19 pandemic in October 2020. Since then, its prevalence has doubled every 6.5 days. It has been associated with a significant increase in COVID-19 infection rates in the UK, partly due to the N501Y mutation. Some evidence shows that this variant has a 40–80% increased transmissibility [52]. 501.v2, 20 h / 501.y.V2 (formerly 20C/501Y.V2) and VOC-20Dec-02 (formerly VOC-202012/02) are tracked by the National Health Department in South Africa. The researchers found that the variant was higher in young people with no underlying health conditions, and it was more likely to cause serious disease in those cases than other variants [53]. Therefore, we believe that the effective measure to solve this problem is the acceleration of vaccine research and development and global sharing.

Secondly, as the case fatality rate reaches the local minimum, the response level of governments of various countries will also decline. This results in a reduction in social distancing and an increase in public activities. Maintaining strict restrictions is the key to solving this problem.

Thirdly, in addition to the government and the virus, some people who do not wear masks in public places and hold public events in violation of restrictions are also important reasons for the repeated fluctuations of the pandemic. We believe that the government should strengthen the publicity and education of health knowledge. Only through the publicity and education of health knowledge can citizens realize the seriousness of COVID-19 and effectively restrain their actions. The use of masks and restrictions on social distance are important evidence of the government’s response. Easing restrictions would mean lowering the current level of epidemic control. First, according to our findings, if the government did not take timely measures to control the COVID-19 outbreak in the previous period, then the government will need to take more measures to control the development of COVID-19 in the current period. Thus, easing restrictions also means the government will have to pay more attention to containing the epidemic in the next period. This provides a powerful incentive for governments to tighten restrictions. Second, despite the restrictions imposed by the government, there may be a situation where citizens are unwilling to comply. It causes weak of the policy executive power. The weak policy executive power will produce the same effect with “doing nothing” policy, namely, it will also lead the government to pay more in the next period for epidemic control costs (in another paper, we used evolutionary game theory in detail discusses the punishment and reward mechanism in the restrictions for citizens and businesses). Therefore, the government must strengthen health science popularization and perfect the reward and punishment mechanism to make citizens abide by the restriction policy.

Compared with the existing literature, our research results extend the empirical evidence on the relationship between government response and COVID-19 in the literature. First, one of our main conclusions is that government response has a dynamic impact on the development of COVID-19, namely, the development of COVID-19 is influenced not only by the level of government response in the current period, but also by the response of the previous period. Existing literature confirms the positive impact of government response on the current development of COVID-19, but there is a lack of research on the dynamic impact. [54] studied how the response of the Ethiopian government suppressed COVID-19 in 2020. They found that containment of COVID-19 could be achieved through a combination of three public health measures: wearing masks, social distancing, and maintaining hygiene. Relaxing any of these three non-drug interventions could lead to a rebound in the number of new cases, they concluded. This rebound also supports our second main finding that government response and COVID-19 control show a cyclical relationship. Easing government restrictions could see a new wave of the epidemic shock. In the Ethiopian context, the most effective public health measure would be for urban populations to wear masks and rural populations to maintain social distance.

The positive role of the government response in suppressing the spread of COVID-19 is also reflected in the research conclusion of [55]. They examined the impact of major interventions in 11 European countries between the start of the COVID-19 pandemic in February 2020 and the start of the lockdown lifting on 4 May 2020. They used the semi-Mechanistic joint Bayesian Hierarchical model to show that the government’s lockdown measures had a huge impact on reducing transmission. They suggest that ongoing interventions should be considered to control the spread of COVID-19. This also supports our main finding: the cyclicality relationship between government response and COVID-19. We also recommend that sustained interventions are limited in curbing the spread of COVID-19 as the same with the suggestions given by [55]. [56] compared the time and severity of government response measures in the United States, Canada, China, Ethiopia, Japan, Kazakhstan, New Zealand, Singapore, South Korea, Vietnam, and Zimbabwe. It was found that in Vietnam, Zimbabwe, New Zealand, South Korea, Ethiopia, and Kazakhstan, early and strict implementation of a set of quarantines for infection, school closures, home isolation and restricted social gatherings reduced both the number of cases and the duration of transmission. In contrast, the United States has rarely implemented aggressive government responses rigorously or early. Their findings suggest that early implementation, consistent implementation, adequate time, and high compliance with government response measures are key factors in reducing the spread of COVID-19. [57] used a Bayesian hierarchical model to estimate the impact of non-pharmaceutical interventions on COVID-19 transmission in 41 countries. Some interventions are more effective than others, as for example the impact of different government restrictions on curbing the spread of COVID-19. This conclusion can make up the deficiency of this paper. We acknowledge that there is no further analysis of specific restrictive policies in this paper. We only analyzed the impact of government responses on the containment of COVID-19 in general. Therefore, the conclusion of [57] ’s study can provide a supplementary explanation for this paper. These findings suggest that by using effective interventions, some countries can control outbreaks without implementing stay-at-home orders. Through the interpretation of the above literature conclusions, our research conclusion added dynamic analysis on their basis. That is, the impact of government response on COVID-19 is intertemporal. This is an extension of the empirical evidence in the current literature.

Similarly, some relevant studies also support another conclusion of our research: government response and the development of COVID-19 show a cyclical relationship. [58] used Fourier transform to analyze the period and synchronicity of time series of COVID-19 infection cases and deaths. They found short and long-term changes in the COVID-19 development. The short period is 7 days. The cyclicality is thought to be caused by community social factors, joint diagnosis, and report cycle. They suggest optimizing infection control strategies by integrating spatial and temporal distances. [59] used spectral density of time series to assess the cycle of the number of coronavirus patients diagnosed each day, thus enabling the government to plan how to allocate resources more effectively. They found that their samples could distinguish between two types of cycles. The first type of cyclical fluctuation is 100 to 300 days. The time of the second type of cyclical fluctuation is about 7 days and the second type of cyclical fluctuation is related to the weekly cycle of population activities. For different countries, the stages of the seven-day fluctuation are consistent. Our study is an empirical extension of these two conclusions. We analyzed the cyclical relationship between government response and the development of COVID-19 based on the COVID-19 cycle.

This paper only analyzes the lag effect of government response on the development of COVID-19. [60] analyzed the causes of time lag through its impact on government expenditure. They argued that once a government has a clear expectation that restrictions will have to be implemented (the option of implementing them now is better than the option of postponing them). Thus, healthcare systems and cost-related variables have a significant impact on response times. Because expectations did not match reality and the government was too confident in its ability to fight the outbreak, it did not immediately implement restrictions. The associated economic costs have raised concerns about high economic costs. Overconfidence and cost concerns have delayed the implementation of restrictions, further increasing the overall cost of fighting the disease (because of the time lag, medical stress and containment costs are higher). Therefore, a rational government, once it is certain that they must impose restrictions, would obviously prefer to anticipate rather than delay.

## 5. Conclusion

Based on the long-term conditions, we used panel data by the dynamic GMM model to solve two research questions: 1. Does the government response affect the development of COVID-19 in the long run? 2. How does the government response affect the development of COVID-19 in the long run? Firstly, we demonstrate through empirical analysis that the government response significantly affects the development of the pandemic. We estimate the relevant parameters through four econometric methods. The results showed that the government response significantly affected the development of the pandemic.

Secondly, the government response has a cyclical effect on the development of COVID-19. As the government response increases, the COVID-19 case fatality rate will decrease, first, to a local minimum point, and will then increase to a local maximum point and finally converge to near 0. At the beginning, the government did not take corresponding measures (only a small number of people were infected at the beginning, so the government did not pay corresponding attention), and the COVID-19 case fatality rate was 0. When the number of infections and deaths increased to a certain level, the government began to realize the seriousness of the pandemic and began to respond. Potentially infected people and already infected people were constantly found, so the case fatality rate suddenly jumped from 0 to a local maximum value. With the continuous increase of government response policies, the case fatality rate decreased, COVID-19 was controlled to a certain extent, and the first wave of the pandemic ended. The mutation of the virus, the loosening of response policies and the lack of social distance among some citizens contributed to the arrival of the second wave. Therefore, the case fatality rate rose from a local minimum point to a local maximum point. Similarly, the case fatality rate will be completely controlled eventually after several rounds of government response (converging to near 0) on a global scale.

As we described in the section 4.4, a large number of facts and literature support our results. Our results are further extended on the basis of existing literature. First, from a dynamic perspective, we once again provide new empirical evidence for government responses to curb the spread of COVID-19. Existing literature provides detailed analysis of how government responses have influenced the development of COVID-19 (some have carefully analyzed the impact of specific government restrictive policies on COVID-19). In addition to confirming the validity of existing literature conclusions, our findings further analyze the intertemporal effects of government response on COVID-19. This is our biggest contribution.

In view of the above conclusions, we believe that the following policies can be implemented. Firstly, since the government response significantly affects the development of the pandemic, the government should improve the response level in the stage of case fatality rate increase. Secondly, the development of the pandemic tends to fluctuate periodically, and countries should be prepared to deal with COVID-19 in the long term. Thirdly, in the absence of exogenous shocks (vaccines), countries should adhere to strict response policies. Fourthly, in addition to the government response, increased publicity on the harm of COVID-19 would strengthen citizens’ capacity for self-restraint. Last but not least, governments should speed up the development of more effective COVID-19 vaccines and strengthen communication and cooperation in vaccine research and development. It is the global sharing of the vaccine that will ultimately allow humans to defeat COVID-19.

We have answered the key question of whether government responses affect the development of the pandemic over the long term, but we have not analyzed what causes the periodic fluctuations of the pandemic. Therefore, future research could be as follows: Firstly, in the intertemporal dynamics, which specific factors in the government response will affect the development of the pandemic? How much do these factors contribute to the development of the pandemic? Secondly, what are the changes after adding the vaccine variable? Vaccine, as a new variable, will greatly reduce (improve) the convergence time (speed) of case fatality rates. Therefore, the vaccine will have a significant impact on pandemic control. It will be an interesting research topic to study the mechanism of vaccine action on the development of the pandemic. Some scholars have made contributions to these issues [57].

Another key issue is that the citizens do not always follow the government’s restrictions. Therefore, whether citizens will comply with the government’s restrictions will be a key research issue. In subsequent studies, we used game theory to analyze under what circumstances citizens will comply with government restrictions and how governments should establish incentives and punishments to ensure compliance. The last important issue is the cost of government response. Sometimes the reason governments do not take sustained, response action is because of the high cost of COVID-19 control. How to balance government expenditure and citizen health is a promising research topic [60].

## Supporting information

S1 Appendix (DOCX)
