# Supplementary material for: The impact of the government response on pandemic control in the long run—A dynamic empirical analysis based on COVID-19
Source: PLoS One. 2022 May 4;17(5):e0267232. doi: 10.1371/journal.pone.0267232 (PMC9067654; doi:10.1371/journal.pone.0267232)
Supplement: S1 Appendix — (DOCX) [file pone.0267232.s001.docx]

**Appendix 1**

Table 1. Panel unit root test results

| Variables and test | Level | | | | | | | | | Fist difference | | | | | | | | |
| --- | --- | --- | --- | --- | --- | --- | --- | --- | --- | --- | --- | --- | --- | --- | --- | --- | --- | --- |
|  | Intercept | | | | Intercept and trend | | | | | Intercept | | | | Intercept and trend | | | | |
|  | Levin, Lin & Chu | Im, Pesaran and Shin W-stat | ADF - Fisher | PP - Fisher | Levin, Lin & Chu | Breitung t-stat | Im, Pesaran and Shin W-stat | ADF - Fisher | PP - Fisher | Levin, Lin & Chu | Im, Pesaran and Shin W-stat | ADF - Fisher | PP - Fisher | Levin, Lin & Chu | Breitung t-stat | Im, Pesaran and Shin W-stat | ADF - Fisher | PP - Fisher |
| $CFR$ | -11.857^***^ (0.00) | -5.842^***^ (0.00) | 56.452^***^ (0.00) | 44.469^***^ (0.00) | -8.444^***^ (0.00) | -3.652^***^ (0.00) | -3.126^***^ (0.00) | 49.949^***^ (0.00) | 54.157^***^ (0.00) | -13.850^***^ (0.00) | -9.746^***^ (0.00) | 96.689^***^ (0.00) | 115.616^***^ (0.00) | -20.070^***^ (0.00) | -3.102^***^ (0.00) | -6.317^***^ (0.00) | 90.946^***^ (0.00) | 121.191^***^ (0.00) |
| $INF$ | -6.819^***^ (0.00) | -4.222^***^ (0.00) | 47.217^***^ (0.00) | 38.680^***^ (0.00) | -6.662^***^ (0.00) | -2.827^***^ (0.00) | -1.907^***^ (0.00) | 33.676^***^ (0.00) | 29.922^**^ (0.02) | -10.872^***^ (0.00) | -5.093^***^ (0.00) | 55.632^***^ (0.00) | 55.969^***^ (0.00) | -10.174^***^ (0.00) | -5.004^***^ (0.00) | -1.647^**^ (0.05) | 36.258^***^ (0.00) | 45.372^***^ (0.00) |
| $GDP\_G$ | -5.067^***^ (0.00) | -2.449^***^ (0.01) | 38.4119^***^ (0.00) | 38.930^***^ (0.00) | -3.883^***^ (0.00) | 0.636 (0.74) | -0.856 (0.12) | 29.433^**^ (0.02) | 49.0649^***^ (0.00) | -11.635^***^ (0.00) | -6.662^***^ (0.00) | 77.105^***^ (0.00) | 120.422^***^ (0.00) | -18.862^***^ (0.00) | 0.034 (0.51) | -4.284*** (0.00) | 68.850^***^ (0.00) | 81.529^***^ (0.00) |
| $(\frac{1}{GOV\_R})\cdot\cos(GOV\_R)$ | -12.833^***^ (0.00) | -8.633^***^ (0.00) | 81.156^***^ (0.00) | 121.500^***^ (0.00) | -11.861^***^ (0.00) | -0.663 (0.25) | -6.176^***^ (0.00) | 62.330^***^ (0.00) | 104.964^**^ (0.04) | -12.882^***^ (0.00) | -9.469^***^ (0.00) | 94.141^***^ (0.00) | 151.073^***^ (0.00) | -6.310^***^ (0.00) | -1.629^**^ (0.05) | -2.770^***^ (0.00) | 52.834^***^ (0.00) | 128.298^**^ (0.00) |
| $CFR(-1)$ | -11.857^***^ (0.00) | -5.842^***^ (0.00) | 56.452^***^ (0.00) | 44.469^***^ (0.00) | -8.444^***^ (0.00) | -3.652^***^ (0.00) | -3.126^***^ (0.00) | 49.949^***^ (0.00) | 54.157^***^ (0.00) | -13.850^***^ (0.00) | -9.746^***^ (0.00) | 96.689^***^ (0.00) | 115.616^***^ (0.00) | -20.070^***^ (0.00) | -3.102^***^ (0.00) | -6.317^***^ (0.00) | 90.946^***^ (0.00) | 121.191^***^ (0.00) |

Notes: ***, ** and * denote significance level at 1%, 5% and 10%, respectively
